# Adenosine-sensitive ventricular tachycardia

**DOI:** 10.1016/j.clinme.2024.100216

**Published:** 2024-05-06

**Authors:** Rahul K. Mukherjee, Magdi M. Saba

**Affiliations:** Department of Cardiology, St George's University Hospitals NHS Foundation Trust, London, UK

**Keywords:** Adenosine, Ventricular tachycardia, Supraventricular tachycardia, Re-entry, Triggered activity, Automaticity

## Case presentation

A 54-year-old male athlete suffered from recurrent episodes of palpitations associated with light-headedness and near syncope. These symptoms were often precipitated by exercise. He had no other medical history and did not report any significant alcohol or caffeine intake. His full blood count, serum electrolytes and thyroid function tests were within normal limits. A 24 h Holter monitor did not reveal any significant arrhythmia. Transthoracic echocardiography and a subsequent cardiac magnetic resonance imaging (CMR) scan revealed a structurally normal heart with no valve disease and normal biventricular function. He therefore underwent an exercise treadmill test and a sustained regular broad complex tachycardia with a left bundle branch block (LBBB) morphology and inferior-axis QRS configuration was induced.

**Fig. 1:**
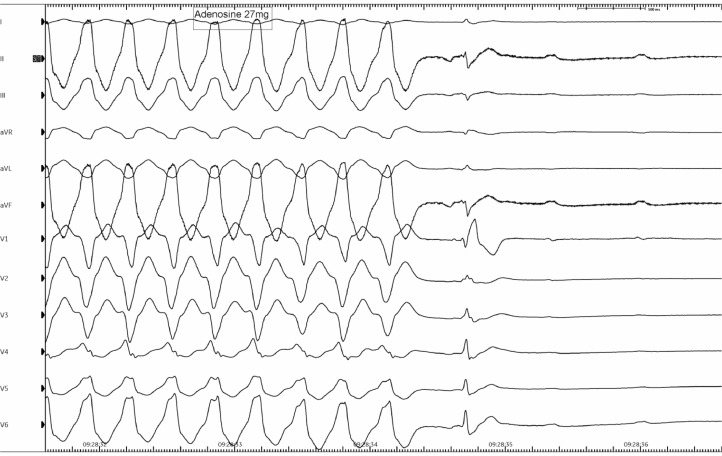
Effect of intravenous adenosine - 12-lead ECG demonstrating termination of broad complex tachycardia with period of AV block.

**Fig. 2:**
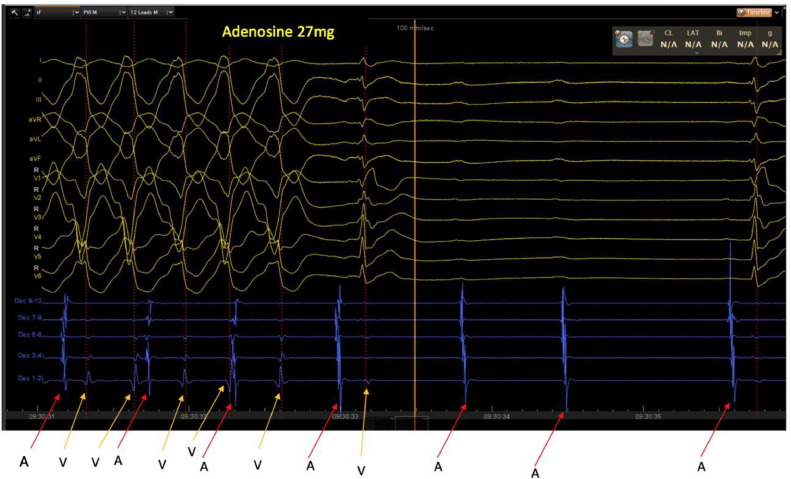
Intracardiac electrogram recordings during intravenous adenosine administration - a decapolar catheter (placed via right femoral vein access in the cardiac catheter laboratory) recording electrical signals is placed in the coronary sinus which records the local atrial signal from five poles (labelled ‘A’) and the far-field ventricular signal (labelled ‘V’). During the broad complex tachycardia, there is clear evidence of A–V dissociation, which proves that the diagnosis is ventricular tachycardia. Following administration of IV adenosine, there is a period of AV block and termination of tachycardia where only local atrial signals are recorded on the decapolar catheter (note P-waves on surface ECG with no QRS complexes during AV block).

**Fig. 3:**
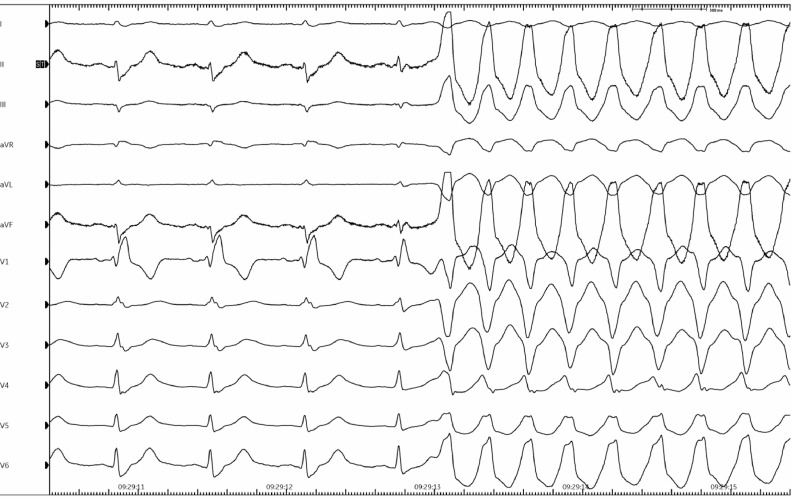
Re-initiation of the same broad complex tachycardia seen on 12-lead ECG after wash-out of adenosine around 40 s later.

An urgent invasive electrophysiology study in the cardiac catheter laboratory was scheduled during which he had catheters placed in the right ventricle (RV) and coronary sinus (CS) via right femoral venous access. With programmed electrical stimulation, the same regular broad complex tachycardia with LBBB morphology and inferior QRS axis was induced. Intravenous administration of adenosine (27 mg) resulted in termination of the tachycardia within 5 s with a period of AV block (Fig. 1). Analysis of the intracardiac electrograms revealed that there was evidence of A–V dissociation on the CS electrograms (with more ventricular beats than atrial beats) during tachycardia, proving that the arrhythmia was ventricular tachycardia (rather than a supraventricular tachycardia with aberrancy or an accessory-pathway mediated tachycardia). A period of AV block followed where only atrial signals were seen on the CS catheter (Fig. 2). Following wash-out of adenosine (after 40 s), there was re-initiation of the broad-complex tachycardia with the same morphology as previously seen (Fig. 3).

Pace-mapping was then performed with an ablation catheter in sinus rhythm to localise the site in the right ventricle which most closely matched the QRS morphologies in each of the 12-leads in tachycardia. A site-of-origin was localised in the antero-septal right ventricular outflow tract (RVOT) with a >99% pace-map match in all 12-leads. This was also corroborated by activation mapping during tachycardia, where the earliest site of activation was found within the area of best pace-map match. Radiofrequency energy was applied to destroy the putative site of tachycardia origin. The patient was discharged home the following day and had a repeat exercise treadmill test 3 months after ablation. No further arrhythmias were inducible and he has not had any further symptoms at follow-up.

## Discussion

The differential diagnosis of a broad complex tachycardia includes supraventricular tachycardia (SVT) with aberrancy, antidromic atrioventricular re-entry tachycardia (AVRT) (accessory-pathway mediated tachycardia) and ventricular tachycardia (VT). Adenosine is an endogenous purine nucleoside that can decrease spontaneous depolarisation in the sinus node and reduce conduction velocity in the atrioventricular (AV) node. It has an ultra-short half-life. Its effect on the atrioventricular (AV) node is the basis for its widespread diagnostic and therapeutic application in patients with supraventricular tachycardias (SVT), either by terminating the arrhythmia, proving its dependence on the AV node or by transiently interrupting AV conduction, thereby unmasking underlying atrial activity and allowing careful analysis of the driving rhythm and reaching a diagnosis.^1^ Conventional teaching states that adenosine has no effect on ventricular tachycardia, but this is only true in the setting of VT, which is due to a re-entrant mechanism. Adenosine can exert an anti-adrenergic response and in ventricular myocardium has an antagonistic effect on catecholamine-stimulated elevations in cAMP.^2^ Although we used 27 mg in our case, doses as low as 6–12 mg have also been shown to produce this response.^2^

There are a cohort of patients with catecholamine-associated, idiopathic ventricular tachycardia (with LBBB morphology and inferior QRS axis) which can terminate with adenosine and also respond to vagal manoeuvres such as carotid sinus massage and Valsalva manoeuvre.^3^ In these patients, the termination of tachycardia with adenosine suggests that the mechanism of the arrhythmia is cAMP-mediated delayed afterdepolarisations (DADs) and triggered activity (not re-entry).^4^ There are several common features shared by such patients including exertion-related sustained or recurrent, non-sustained VT, the presence of a structurally normal heart and an apparent predilection for sites of origin to localise to the free wall of the pulmonary infundibulum or at the septal insertion in the RVOT.^2^ Catheter ablation is a safe and highly efficacious therapeutic option for these patients, with success rates approaching >90% and a low risk of complications.^5^

## Conclusion

Idiopathic right ventricular outflow tract arrhythmias caused by cAMP-mediated triggered activity can be adenosine sensitive. In patients with no history of structural heart disease or an otherwise structurally normal heart presenting with a broad complex tachycardia with a LBBB morphology and inferior QRS axis, the administration of intravenous adenosine can be used to terminate the tachycardia and provide a clue to the mechanism - even when the diagnosis is ultimately ventricular tachycardia.

## Author contributions

RKM and MMS wrote and reviewed the manuscript.

## Patient consent

The patient gave written informed consent for the publication of this case report.

## Declaration of competing interest

The authors declare that they have no known competing financial interests or personal relationships that could have appeared to influence the work reported in this paper.
